# Locomotor-State Dependence of Neuromodulation During Avian Free Flight

**DOI:** 10.3390/ani16203213

**Published:** 2026-10-13

**Authors:** Zhuo Tang, Suchen Li, Mengmeng Li, Zhigang Shang

**Affiliations:** 1 School of Electrical and Information Engineering, Zhengzhou University, Zhengzhou 450001, China; tz@gs.zzu.edu.cn (Z.T.); suchenli@gs.zzu.edu.cn (S.L.); limengmeng@zzu.edu.cn (M.L.); 2 Henan Key Laboratory of Brain Science and Brain-Computer Interface Technology, Zhengzhou 450001, China

**Keywords:** avian neuromodulation, locomotor state, state dependence, free flight

## Abstract

Complex avian locomotor behaviors depend on the coordinated operation of neural processes involved in sensory perception, decision-making, and motor execution. Exogenous neural stimulation can alter motor output by intervening in neural processes, but its effects may depend on the current locomotor state. Here, we investigated the locomotor-state dependence of formatio reticularis medialis mesencephali (FRM) microstimulation in freely flying pigeons. Individualized stimulation parameters were screened under stable laboratory conditions and kept constant during free-flight experiments. Flight-state features before and during stimulation were extracted, and state–response models were constructed to quantify their associations with stimulation-evoked turning responses. Results showed that flight state during stimulation was more closely associated with turning responses than pre-stimulation state, with acceleration during stimulation making the largest contribution. When acceleration during stimulation was above the individual mean, turning-response dispersion decreased by 32.92%. In addition, pre-stimulation angular velocity was positively associated with acceleration during stimulation, indicating that the pre-stimulation locomotor state contains information related to the consistency of subsequent stimulation-evoked responses. These findings demonstrate that the effects of FRM microstimulation are locomotor-state dependent and provide a basis for identifying appropriate neuromodulation timing and improving response stability under complex locomotor conditions.

## Simple Summary

This study investigated whether the effects of neuromodulation in freely flying pigeons depend on their current locomotor state. Using individualized electrical stimulation of a midbrain motor region, we examined the relationships between flight states before and during stimulation and the resulting turning responses. Flight state during stimulation was more closely associated with turning responses than pre-stimulation state, with acceleration showing the strongest relationship. When acceleration during stimulation was above the individual average, turning-response variability decreased by 32.92%. Pre-stimulation angular velocity was also related to subsequent acceleration during stimulation. These findings demonstrate the locomotor-state dependence of neuromodulation and provide a basis for identifying more appropriate stimulation timing to improve the stability of neuromodulation.

## 1. Introduction

Complex avian behaviors such as long-distance homing, obstacle avoidance, and flight require the coordinated operation of neural processes involved in sensory perception, decision-making, and motor execution to generate appropriate behavioral responses to a continuously changing environment [1,2]. Understanding the neural mechanisms underlying information processing and motor control is essential for explaining how avian behavior is generated and for developing neuromodulatory approaches. Previous studies of visual perception [3], spatial cognition [4], and task-related decision-making [5] have revealed how birds acquire external information and process it through neural encoding, integration, and transmission to generate neural representations and behavioral outputs. These findings indicate that avian locomotor behavior does not arise from a single neural process but instead emerges from the coordinated interaction of sensory input, neural information processing, and motor execution. Building on this understanding, exogenous intervention in specific neural processes to modify neural activity and thereby influence behavioral output has become an important approach for extending research from understanding neural mechanisms to actively modulating animal behavior.

The use of exogenous neural stimulation to modulate locomotor behavior is, in essence, an intervention in the animal’s ongoing neural information-processing system. The basic principle is to select a brain region and stimulation site according to the neural function involved in the target behavior and to alter local neural activity so that stimulation-evoked activity engages existing neural and motor-control pathways, ultimately producing a corresponding behavioral response [6]. Rather than directly controlling individual muscles or joints, this approach relies on the animal’s own neural and motor systems to translate local neural intervention into coordinated whole-body behavior. Based on this principle, stimulation of different functional brain regions has been used to evoke fear- and defense-related responses [7], reward-related responses [8], and directed locomotion and turning [9], and these approaches have increasingly been applied to the modulation of avian locomotion.

Different avian brain regions participate in sensory, emotional, and motor processes, and therefore influence locomotor behavior through distinct mechanisms, giving rise to different neuromodulatory approaches. The nucleus dorsalis intermedius ventralis anterior (DIVA) participates in the transmission and processing of somatosensory information [10], and electrical stimulation on either side can induce directional turning and has been used to control flight direction in open space [6]. The stratum griseum periventriculare (SGP) is associated with innate fear responses in birds [11], and stimulation of this region can evoke avoidance behavior and can be used for directional control during free flight [7]. The posterior pallial amygdala (PoA) participates in emotion-related neural processing within broader forebrain–midbrain circuits. Electrical stimulation of the PoA has been reported to evoke multiple forms of motor response in pigeons rather than a single stereotyped behavioral output [12]. However, prolonged intervention in sensory- or emotion-related neural processes may be accompanied by maladaptive physiological responses [13]. Neural structures more directly involved in motor control have therefore attracted increasing attention. Neural activity in the medial reticular formation (FRM) is closely associated with avian flight state, with distinct activity patterns observed across different flight states [14]. Exogenous electrical stimulation of the FRM can alter neural oscillatory activity and is accompanied by directional turning behavior [15]. Further studies have shown that unilateral FRM stimulation can reliably induce turning responses, that the magnitude of these responses varies with stimulation parameters, and that FRM stimulation can support more complex control of flight direction [13,16,17]. However, stimulation-evoked neural activity does not act against an isolated neural background. During ongoing movement, the nervous system continuously receives sensory feedback and processes information related to the current locomotor state, while stimulation-evoked activity interacts with these ongoing neural processes in shaping motor output [18]. Consequently, even when the stimulation target and parameters remain unchanged, the neural context in which stimulation is delivered can differ across locomotor states, potentially resulting in different neuromodulatory effects [19]. Characterizing this locomotor-state dependence and using such state information to identify more appropriate times for neuromodulation are therefore important for improving the stability and predictability of neuromodulation under complex locomotor conditions.

To address this issue, we investigated FRM stimulation-evoked turning responses during free flight and examined their relationship with the locomotor state under fixed stimulation conditions. By separately characterizing locomotor states before and during stimulation, we examined how states at these two stages were associated with the magnitude and consistency of stimulation-evoked turning responses and further analyzed the relationship between pre-stimulation and during-stimulation states. Together, these analyses were designed to characterize the locomotor-state dependence of FRM neuromodulation during free flight and to provide a basis for identifying appropriate neuromodulation timing and improving the stability of neuromodulation under complex locomotor conditions.

## 2. Materials and Methods

### 2.1. Animals and Surgery

**Figure 1:**
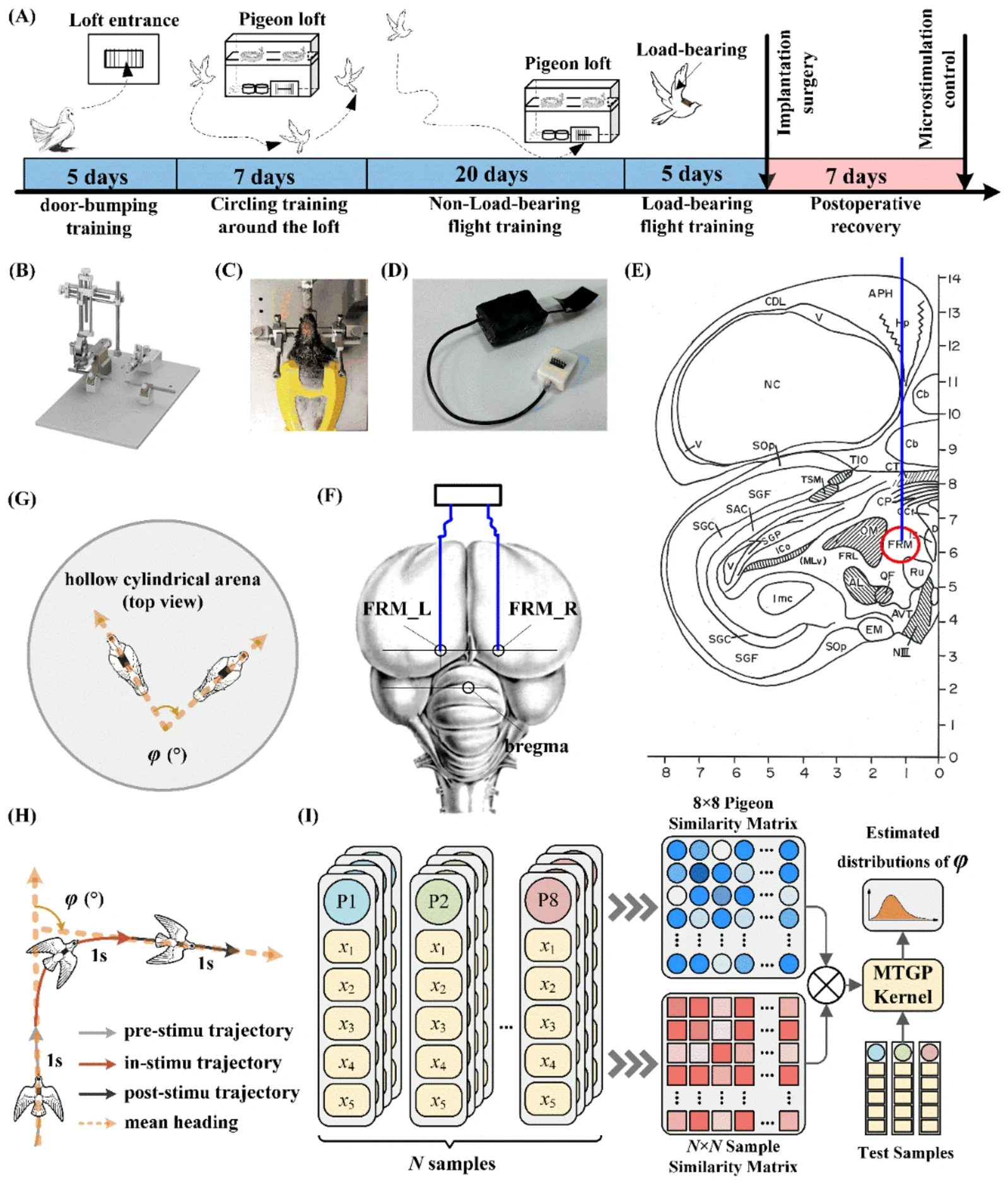
Experimental procedures and state–response modeling framework. (**A**) Pre-flight training; (**B**,**C**) stereotaxic setup and electrode implantation; (**D**) miniature neural stimulation and motion-recording device; (**E**,**F**) FRM target and electrode implantation site; (**G**,**H**) laboratory and free-flight turning-response definitions; (**I**) state–response modeling framework.

Eight healthy adult homing pigeons (*Columba livia*) were used in this study. The pigeons were laboratory-bred homing pigeons from a breeding colony in Zhengzhou, Henan Province, China, where they had been routinely raised under standard loft conditions. In the present study, homing ability refers to the ability of the pigeons to return to their home loft after release from progressively increasing distances. All pigeons had been housed in a loft since hatching under a natural light–dark cycle, with free access to water. They were fed a commercially purchased mixture of corn and mixed grains obtained from local commercial feed suppliers in Zhengzhou, China, once daily between 16:00 and 17:00. Body mass remained stable at 430–460 g throughout the experiments. Before formal testing, all pigeons sequentially underwent loft acclimation, circling flights around the loft, long-distance release training, and load-acclimation training, as shown in Figure 1A. The release distance was gradually increased from 1 to 10 km, and load training was performed using a mock device with a mass comparable to that of the experimental apparatus. After training, all eight pigeons showed stable homing-flight performance during repeated releases and were included in the subsequent experiments.

After flight training, bilateral FRM stimulation electrodes were implanted in all eight pigeons. Food was withheld for 24 h before surgery while water remained freely available. General anesthesia was induced by intraperitoneal injection of 3% sodium pentobarbital solution at 0.15 mL/100 g body weight and supplemented with local anesthesia by subcutaneous injection of 0.5 mL of 2% lidocaine hydrochloride into the cranial surgical area. After deep anesthesia was achieved, each pigeon was secured in a stereotaxic apparatus. Based on a stereotaxic atlas of the pigeon brain, the bilateral FRM coordinates were set at 4.25 mm anteroposteriorly, ±2.0 mm mediolaterally, and 8.0 mm dorsoventrally, as shown in Figure 1B,C,E,F. Nickel–chromium stainless-steel wires with a diameter of 100 μm were implanted bilaterally as stimulation electrodes, and the implantation sites were sealed with silicone gel. The electrode assembly and reference electrode were fixed with skull screws and dental resin and connected to a custom electrode base. A 7-day postoperative recovery period was allowed. During recovery, feeding behavior and spontaneous locomotion were monitored daily. Before resuming the experiments, homing-flight behavior was assessed through release flights, and only pigeons showing feeding, locomotor, and homing-flight performance comparable to their preoperative condition were included in the subsequent experiments. After completion of all experiments, electrode locations were verified by electrolytic lesions and histological examination.

### 2.2. Individualized Stimulation Parameter Screening

To minimize the influence of variations in stimulation parameters on the analysis of free-flight state–response relationships, FRM stimulation parameters were screened individually for each pigeon under laboratory ground conditions in which the locomotor state was relatively stable. The aim was to select, for each pigeon, a stimulation amplitude that evoked an approximately 90° directional turning response under relatively stable ground conditions. Compared with outdoor free flight, both the pigeons’ locomotor state and the external environment were relatively stable under ground conditions, reducing the effects of spontaneous movement and environmental variation on the evaluation of stimulation responses. After postoperative recovery, each pigeon was placed in a cylindrical arena 1 m in diameter and stimulated while relatively stationary. The change in body orientation from stimulation onset to stimulation offset was defined as the ground turning response, as shown in Figure 1G.

FRM microstimulation was delivered locally through the implanted 100 μm fine-wire electrodes using charge-balanced, cathodic-leading biphasic rectangular pulses with equal pulse widths in the positive and negative phases [20,21,22,23]. Based on previous studies, stimulation frequency, pulse width, and duration were fixed at 60 Hz, 2 ms, and 1 s, respectively [9,13,16]. Because the turning response evoked by FRM stimulation is approximately related to stimulation amplitude within a certain range [16], only stimulation amplitude was varied during individualized screening while all other stimulation parameters were held constant. To reduce possible response attenuation caused by repeated stimulation over short intervals, consecutive stimulations were separated by at least 10 s, and each amplitude was tested 10 times. The stimulation amplitude subsequently used in the free-flight experiment was selected for each pigeon according to its ground turning responses across amplitudes. Once the parameters had been selected, stimulation frequency, pulse width, duration, and amplitude were kept constant for each individual throughout the outdoor experiments, allowing changes in FRM stimulation-evoked turning responses across different free-flight states to be examined under controlled stimulation conditions.

### 2.3. Outdoor Free-Flight Stimulation Experiment

To investigate the relationship between locomotor state and FRM stimulation-evoked turning responses during free flight, outdoor free-flight stimulation experiments were conducted using the individualized parameters screened under laboratory ground conditions. For each pigeon, the stimulation parameters remained unchanged throughout the outdoor experiments, while flight states before and during stimulation and the corresponding turning responses were recorded synchronously. To reduce interference from spontaneous turning, stimulation was delivered only when the flight trajectory was approximately straight. Consecutive stimulations were separated by at least 10 s to reduce possible response attenuation caused by short-interval repeated stimulation. All outdoor free-flight experiments were conducted in clear weather with wind speeds below 2 m/s to minimize the effects of environmental variability on flight state and stimulation response. Experiments were scheduled before the pigeons’ normal feeding time to maintain homing motivation. During transport from the loft to the release site, the transport cage was covered with black cloth to reduce transport-related stress. After arrival, pigeons were allowed 15 min to acclimate to the environment before release. A custom miniature neural stimulation and motion-recording device was used, as shown in Figure 1D. The device measured 30 mm × 20 mm × 13 mm and weighed approximately 15.8 g. It integrated a stimulation-pulse module, an inertial measurement unit (IMU), and a global positioning system (GPS) module, enabling synchronized acquisition and storage of stimulation output, flight attitude, and flight-trajectory data. The main specifications of the hardware modules are listed in Table 1.

The recorded flight data were processed according to the method of Nagy et al. [24]. Flight trajectories recorded as latitude and longitude were first converted to the Universal Transverse Mercator (UTM) coordinate system. Moving-average filters with window lengths of 6 and 10 were then applied to the flight-trajectory and attitude data, respectively, to reduce measurement noise while preserving short-term movement changes. After preprocessing, flight-state features were extracted from the trajectory and attitude data for subsequent state–response relationship analysis [13,25,26]. Pre-stimulation and during-stimulation flight states were characterized separately, and the FRM stimulation-evoked turning angle was used as the behavioral response variable, as defined below.

Stimulation-evoked turning angle (φ): To account for flight inertia during free flight and the continuation of flight maneuvers after stimulation ended, the mean flight heading during the 1 s before stimulation onset was used as the reference heading. The angular change between the mean flight heading during the 1 s after stimulation offset and the reference heading was calculated and defined as the FRM stimulation-evoked turning angle, as shown in Figure 1H. This variable quantified the change in flight direction associated with each stimulation trial and served as the behavioral response measure in the subsequent state–response analysis.

Pre-stimulation and during-stimulation flight acceleration ($A_{p}$, $A_{m}$): Flight acceleration was used to characterize acceleration-related locomotor states before and during stimulation and was defined as the mean magnitude of triaxial acceleration measured by the IMU within the corresponding time window:

(1) $$A=\frac{1}{T}\sum_{t=1}^{T}\sqrt{a_{x}^{2}\left(t\right)+a_{y}^{2}\left(t\right)+a_{z}^{2}\left(t\right)},$$

where $a_{x}$, $a_{y}$, and $a_{z}$ denote the acceleration measurements along the three axes of the inertial measurement unit, respectively.

Pre-stimulation and during-stimulation flight angular velocity ($G_{p}$, $G_{m}$): Because changes in angular velocity can partly reflect attitude variation during flight, flight angular velocity was used to characterize the attitude-change intensity before and during stimulation. It was defined as the mean magnitude of triaxial angular velocity measured by the inertial measurement unit within the corresponding time window:

(2) $$G=\frac{1}{T}\sum_{t=1}^{T}\sqrt{g_{x}^{2}\left(t\right)+g_{y}^{2}\left(t\right)+g_{z}^{2}\left(t\right)},$$

where $g_{x}$, $g_{y}$ and $g_{z}$ denote the angular velocity measurements along the three IMU axes, respectively.

Pre-stimulation and during-stimulation flight velocity ($V_{p}$, $V_{m}$): Flight velocity reflects the current flight-movement level. In this study, the mean differential velocity calculated from the flight trajectory within the corresponding pre-stimulation and during-stimulation time windows was used as the flight-velocity feature.

### 2.4. State–Response Relationship Modeling

To quantitatively characterize the relationship between free-flight locomotor state and FRM stimulation-evoked turning responses, state–response relationship models were constructed separately using flight-state features before and during stimulation, and the correspondence between state information at the two stages and the turning response was compared. Because the stimulation parameters were held constant for each pigeon during the outdoor free-flight experiments, the models were primarily used to analyze how changes in locomotor state under fixed stimulation conditions were associated with changes in the stimulation-evoked turning response. Flight velocity, acceleration, and angular velocity before or during stimulation were used as model inputs, and the FRM stimulation-evoked turning angle was used as the output. Because correlations may exist among locomotor-state features, variance inflation factor (VIF) analysis was performed separately for the input features at the two stages before modeling to test for multicollinearity and reduce the influence of feature redundancy on relationship quantification and model interpretation.

Pigeons differ in baseline flight state, electrode implantation location, and individual physiological characteristics. Accordingly, the relationship between locomotor state and stimulation-evoked turning response may also vary across individuals. In addition, outdoor free-flight stimulation experiments are difficult to perform, and only a limited number of valid stimulation samples can be obtained from each pigeon. Data from a single pigeon alone may therefore be insufficient to characterize its state–response relationship robustly. For a conventional Gaussian process model fitted separately to each pigeon, the covariance structure, kernel hyperparameters, and observation noise must be estimated solely from that individual’s limited observations, which can lead to less stable parameter estimation and reduced generalization under small per-animal sample sizes. We consequently used a multi-task Gaussian process (MTGP) [27,28], treating the data from each pigeon as an independent task. A task-related kernel simultaneously captured patterns shared across individuals and individual-specific differences, thereby using cross-individual information to improve the stability of relationship modeling under limited-sample conditions. The model framework is shown in Figure 1I.

For m pigeons, the flight-state feature vectors X and the corresponding observed stimulation-evoked turning angles κ can be expressed as

(3) $$X=\left\{x_{i}^{j}|\;j=1,2,\ldots ,m;i=1,2,\ldots ,N^{j}\right\},\;\kappa =\left\{\varphi_{i}^{j}|\;j=1,2,\ldots ,m;i=1,2,\ldots ,N^{j}\right\},$$

where j denotes the index of the pigeon, and $N^{j}$ denotes the number of samples corresponding to the j-th pigeon. To distinguish different individuals, an individual index $l_{j}=j$ was introduced as an additional input to the model, and the covariance function was defined as

(4) $$k_{MTGP}\left(x,x\prime ,l,l\prime \right)=k_{c}\left(l,l\prime \right)\times k_{t}\left(x,x\prime \right),$$

where $k_{c}$ describes the correlation between different pigeons, and $k_{t}$ represents the correlation between different locomotor-state samples. In this study, the anisotropic squared exponential (RBF) kernel was adopted to describe the covariance between flight-state features (kernel-ablation results are provided in Table S3). Accordingly, the covariance matrix of the state–response relationship model can be written as

(5) $$K_{MTGP}\left(X,l,\theta_{c},\theta_{t}\right)=K_{c}\left(l,\theta_{c}\right)⨂K_{t}\left(X,\theta_{t}\right),$$

where ⨂ denotes the Kronecker product, $K_{c}\in R^{m\times m}$ describes the correlation structure across pigeons, and $K_{t}\in R^{N^{j}\times N^{j}}$ characterizes the covariance structure among flight-state samples. $\theta_{c}$ and $\theta_{t}$ denote the corresponding model parameters. To ensure that the covariance matrix $K_{c}$ is positive semi-definite and satisfies Mercer’s condition, Cholesky decomposition was adopted:

(6) $$K_{c}=LL^{⊺},\;L=\left[\begin{array}{cccc} \theta_{c,1} & 0 & \ldots & 0\\ \theta_{c,2} & \theta_{c,3} & \ldots & 0\\ \vdots & \vdots & \ddots & \vdots \\ \theta_{c,k-m+1} & \theta_{c,k-m+2} & \ldots & \theta_{c,k} \end{array}\right],\;k=\sum_{i=1}^{m}i,$$

The model parameters $\theta_{c}$, $\theta_{t}$ and the observation-noise parameters were estimated by minimizing the negative log marginal likelihood. Given a flight-state input $\left\{x_{*},l_{*}\right\}$, the corresponding stimulation-evoked turning angle was characterized by the conditional predictive distribution $p\left(\kappa_{*}|X,l,\kappa ,x_{*},l_{*}\right)$, from which the predictive mean and associated uncertainty were obtained.

To evaluate how well different models characterized the flight-state–stimulation-response relationship, MTGP was compared with a standard Gaussian process (GP) and a single-task Gaussian process (STGP). Specifically, the GP model jointly modeled all outdoor free-flight stimulation data without explicitly distinguishing individual differences, whereas the STGP model fitted a separate model using only the data from each individual pigeon. In addition, because $\varphi_{i}^{j}=f^{j}\left(x_{i}^{j}\right)+\varepsilon_{i}^{j}$, where the observation noise satisfies $\varepsilon_{i}^{j}\sim N\left(0,\sigma_{j}^{2}\right)$, two MTGP variants were constructed to examine how different observation-noise assumptions affected characterization of both the stimulation-evoked turning response and its variability. The shared-noise multi-task Gaussian process (S-MTGP) assumed the same observation noise for all pigeons, i.e., $\sigma_{j}^{2}=\sigma^{2},\;\forall j$; the task-specific-noise multi-task Gaussian process (T-MTGP) allowed each individual to have an independent observation-noise term, namely, $\sigma_{j}^{2}$ varies with j, thereby representing between-individual differences in stimulation-response variability. All models were trained and evaluated using five-fold cross-validation. For each pigeon, the samples were divided into five subsets such that every training and test fold contained data from all individuals. Because FRM stimulation-evoked turning responses showed appreciable variability, model evaluation needed to consider not only goodness of fit to the observed turning angles but also the ability to represent response uncertainty. Negative log likelihood (NLL) was therefore used as the primary evaluation metric; a lower NLL indicates more appropriate characterization of both the stimulation-evoked turning angle and its uncertainty.

### 2.5. Model Interpretation and Key Flight-State Feature Analysis

To further analyze the relationships between flight-state features and FRM stimulation-evoked turning responses, Shapley additive explanation (SHAP) was used to interpret the fitted state–response models. SHAP is a post hoc model-interpretation method based on game theory that quantifies the relative importance of input features by calculating their marginal contributions to model output [29,30]. For each stimulation trial, a SHAP value was calculated for each locomotor-state feature, and feature importance was quantified as the mean absolute SHAP value across all samples. A larger absolute SHAP value indicates a greater contribution of that feature to variation in model output. The SHAP value $\phi_{i}$ for feature i is calculated as follows:

(7) $$\phi_{i}=\sum_{S\subseteq N\setminus \{i\}}\frac{|S|!\cdot \;(|N|-|S|-1)!}{|N|!}\left[\int \left(S\cup \{i\}\right)-\int \left(S\right)\right],$$

where N denotes the set of all input features, and S denotes a subset of features that does not include feature i. This formulation considers all possible feature combinations and evaluates the marginal contribution of feature i across different subsets, thereby yielding its SHAP value $\phi_{i}$. In addition, the mean output of the state–response relationship model was used as the quantity to be explained in the SHAP analysis. By examining the relationship between the values of key flight-state features and their corresponding SHAP values, the direction and variation of their contributions to the model output across different value ranges were further analyzed [31].

## 3. Results

### 3.1. Variability in Stimulation-Evoked Turning Responses

**Figure 2:**
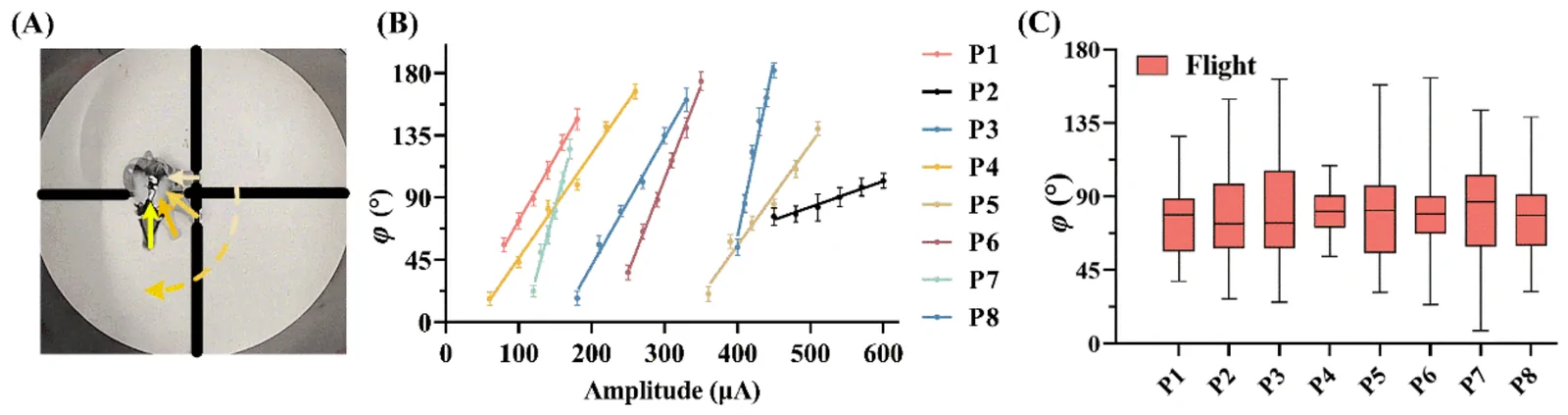
Stimulation parameter selection and variability in stimulation-evoked turning responses. (**A**) Schematic illustration of the turning response under laboratory ground conditions, where the straight arrow indicates the body orientation of the pigeon and the curved arrow indicates the turning direction following stimulation; (**B**) relationship between stimulation amplitude and stimulation-evoked turning angle (φ); (**C**) distribution of stimulation-evoked turning angles across pigeons during outdoor free flight.

Individualized stimulation parameters were screened for eight pigeons under laboratory ground conditions. Stimulation frequency, pulse width, and duration were held constant, while stimulation amplitude was varied when the pigeons were in a relatively stable locomotor state, as shown in Figure 2A. Turning responses at different stimulation amplitudes are shown in Figure 2B. Under relatively stable locomotor conditions, stimulation amplitude showed an overall positive linear relationship with stimulation-evoked turning angle. Although the effective amplitude range differed across individuals, all pigeons showed an increase in turning angle as stimulation amplitude increased. Based on this relationship, an amplitude that reliably induced an approximately 90° turning response was selected for each pigeon, and the resulting individualized stimulation parameters were kept constant during subsequent outdoor free-flight experiments. The stimulation amplitudes and numbers of stimulation trials for each pigeon are provided in Table S1. Under outdoor free-flight conditions, turning responses evoked by fixed stimulation parameters showed substantially greater dispersion, with a standard deviation of 24.98°, as shown in Figure 2C. Thus, considerable variation in turning responses remained during free flight even when stimulation conditions were unchanged.

### 3.2. State Dependence of Turning Responses

#### 3.2.1. Pre- vs. During-Stimulation State–Response Relationships

To determine whether stimulation-evoked turning responses during free flight were related to flight state, separate relationship models were constructed for pre-stimulation and during-stimulation flight states while keeping the stimulation parameters constant for each individual. Flight velocity, acceleration, and angular velocity at each stage were used as inputs, and the stimulation-evoked turning angle was used as the output. The multicollinearity results for the flight-state features are presented in Table S2. The maximum variance inflation factors (VIFs) for the pre-stimulation and during-stimulation feature sets were 1.36 and 1.59, respectively, both below 5 [32], indicating no substantial multicollinearity among the input features and supporting their use in the subsequent state–response analyses.

**Figure 3:**
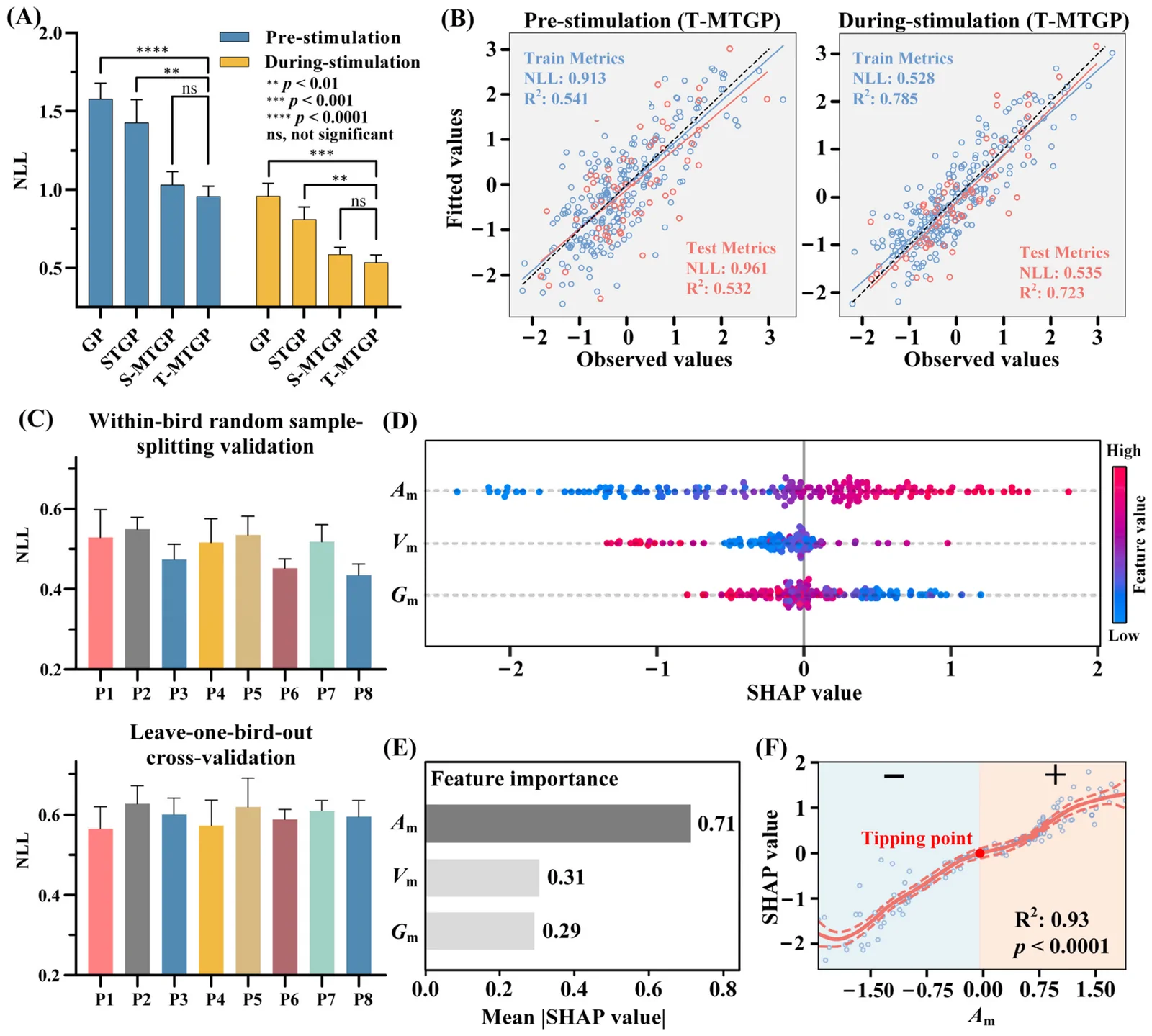
State–response relationships and key during-stimulation feature. (**A**) Model performance; (**B**) observed versus predicted turning responses; (**C**) individual NLLs under within-bird and leave-one-bird-out validation; (**D**) SHAP-value distribution; (**E**) feature importance; (**F**) GAM-fitted relationship between during-stimulation acceleration and its SHAP value.

Figure 3A–C compares the performance of different models in characterizing the relationships between flight state and turning response before and during stimulation. As shown in Figure 3A, T-MTGP achieved the lowest NLL for both state–response relationships. For the pre-stimulation model, the NLL of T-MTGP was 39.24% and 32.87% lower than that of GP and STGP, respectively; for the during-stimulation model, it was 43.75% and 33.33% lower, respectively. These results indicate that incorporating cross-individual information improves quantification of the state–response relationship when sample sizes are limited. T-MTGP showed only a small difference in overall fitting performance relative to S-MTGP, but its individual-specific observation-noise terms additionally captured differences in stimulation-response variability among pigeons.

Comparison of the two state–response relationships further showed that flight state during stimulation corresponded more closely to the stimulation-evoked turning response. Within the T-MTGP framework, the during-stimulation model had an NLL of 0.535, 44.27% lower than that of the pre-stimulation model. Consistent with this result, Figure 3B shows that predictions from the during-stimulation model were generally closer to the ideal agreement line, whereas the pre-stimulation model showed greater scatter. These findings indicate that variation in the stimulation-evoked turning response is more closely related to the flight state expressed during stimulation than to the free-flight state present before stimulation.

Figure 3C further shows the NLL distribution of the during-stimulation T-MTGP model across the eight pigeons, evaluated using both within-bird random-split validation and leave-one-bird-out cross-validation. Under both validation schemes, NLL values remained relatively low for all individuals and varied only modestly across pigeons, further indicating that the relationship between flight state during stimulation and the stimulation-evoked turning response was reasonably stable across individuals.

#### 3.2.2. Key During-Stimulation State Feature

Because flight state during stimulation showed a stronger correspondence with the stimulation-evoked turning response, SHAP analysis was used to quantify the relative contributions of individual flight-state features and identify the state feature most closely associated with variation in the turning response, as shown in Figure 3D–F.

Figure 3D shows the distribution of SHAP values for the flight-state features during stimulation, and Figure 3E shows the corresponding mean absolute SHAP values. During-stimulation acceleration $A_{m}$ had the highest mean absolute SHAP value (0.71), whereas flight velocity $V_{m}$ and angular velocity $G_{m}$ had values of 0.31 and 0.29, respectively. In addition, $A_{m}$ showed a wider range of SHAP values, indicating a stronger correspondence between changes in this feature and changes in model output. To further examine the relationship between $A_{m}$ and turning response, the value of $A_{m}$ for each stimulation sample was paired with its corresponding SHAP value and fitted using a generalized additive model, as shown in Figure 3F. The relationship between $A_{m}$ and its SHAP value was approximately monotonically increasing ($R^{2}=0.93$, p<0.0001). When $A_{m}$ was below approximately −0.04, the corresponding SHAP values were predominantly negative; as $A_{m}$ increased beyond this transition point, the SHAP values became positive and continued to increase. These results indicate that, among the flight-state features during stimulation, acceleration $A_{m}$ was most closely associated with variation in the stimulation-evoked turning response.

### 3.3. Association Between Pre- and During-Stimulation States

The preceding results showed that acceleration during stimulation $A_{m}$ was the locomotor-state feature most closely associated with changes in the stimulation-evoked turning response. To determine whether variation in $A_{m}$ was related to the pre-stimulation free-flight state, a pre-to-during stimulation state relationship model was constructed using pre-stimulation flight velocity $V_{p}$, acceleration $A_{p}$, and angular velocity $G_{p}$ as inputs and during-stimulation acceleration $A_{m}$ as the output. This model was used to quantify the correspondence between pre-stimulation locomotor state and the key state during stimulation.

**Figure 4:**
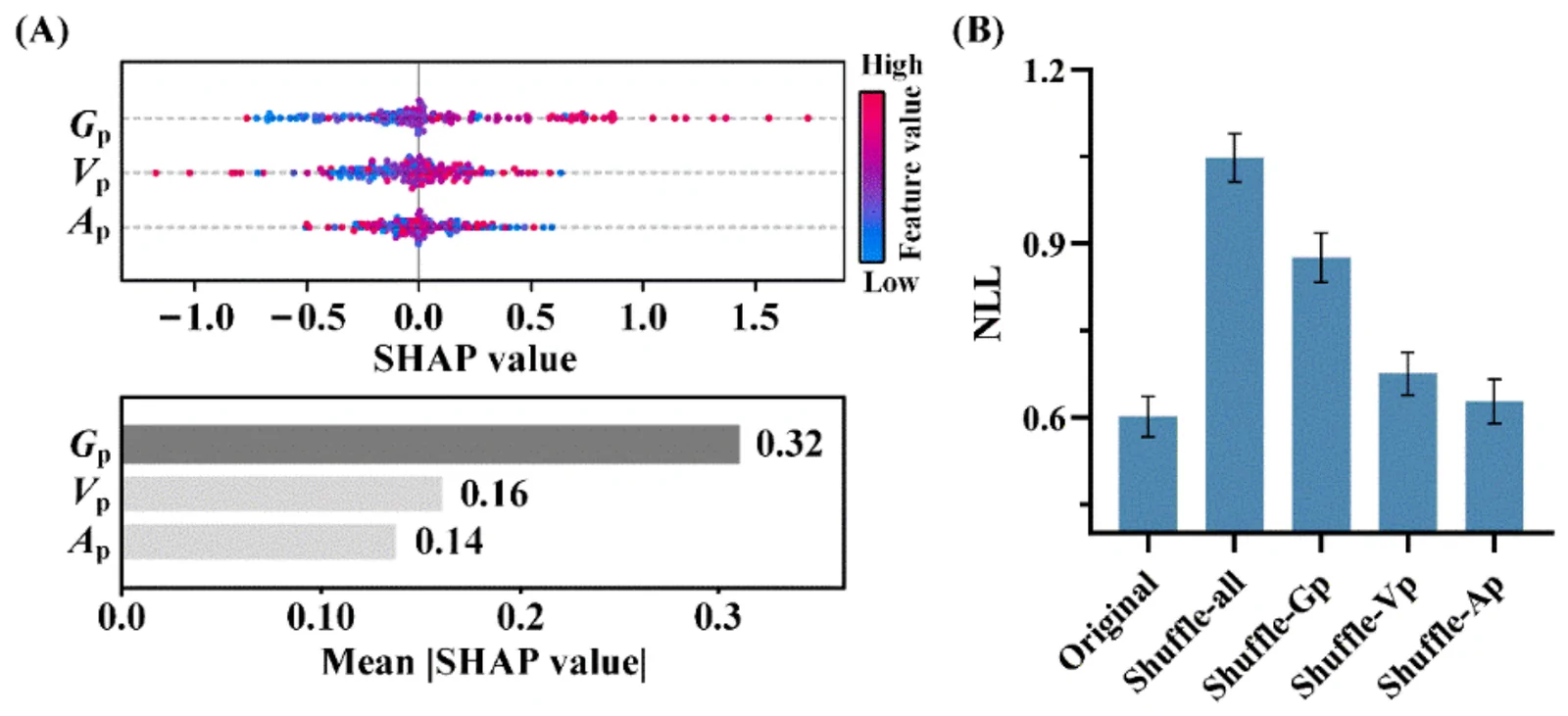
Association of pre-stimulation flight-state features with during-stimulation acceleration. (**A**) SHAP value distributions and importance of pre-stimulation flight-state features for $A_{m}$; (**B**) feature-permutation validation of the relationship between pre-stimulation flight-state features and $A_{m}$.

Figure 4A shows the SHAP values and mean absolute SHAP values of the three pre-stimulation flight-state features. Pre-stimulation angular velocity $G_{p}$ had the highest mean absolute SHAP value (0.32), followed by flight velocity $V_{p}$ (0.16) and acceleration $A_{p}$ (0.14). These results indicate that variation in the key during-stimulation acceleration state $A_{m}$ was not independent of the preceding free-flight state and that pre-stimulation angular velocity $G_{p}$ showed the strongest correspondence with changes in $A_{m}$.

To further validate this relationship, permutation analysis was performed for each pre-stimulation state feature, as shown in Figure 4B. The original model had an NLL of 0.602, which increased by 74% when all pre-stimulation state features were permuted simultaneously. Under single-feature permutation, permuting $G_{p}$ produced the largest increase in NLL, exceeding the increases caused by permuting $V_{p}$ or $A_{p}$. The SHAP and permutation analyses therefore consistently identified pre-stimulation angular velocity $G_{p}$ as the pre-stimulation flight-state feature most closely related to the key during-stimulation acceleration state $A_{m}$.

### 3.4. State Conditions for Turning-Response Consistency

**Figure 5:**
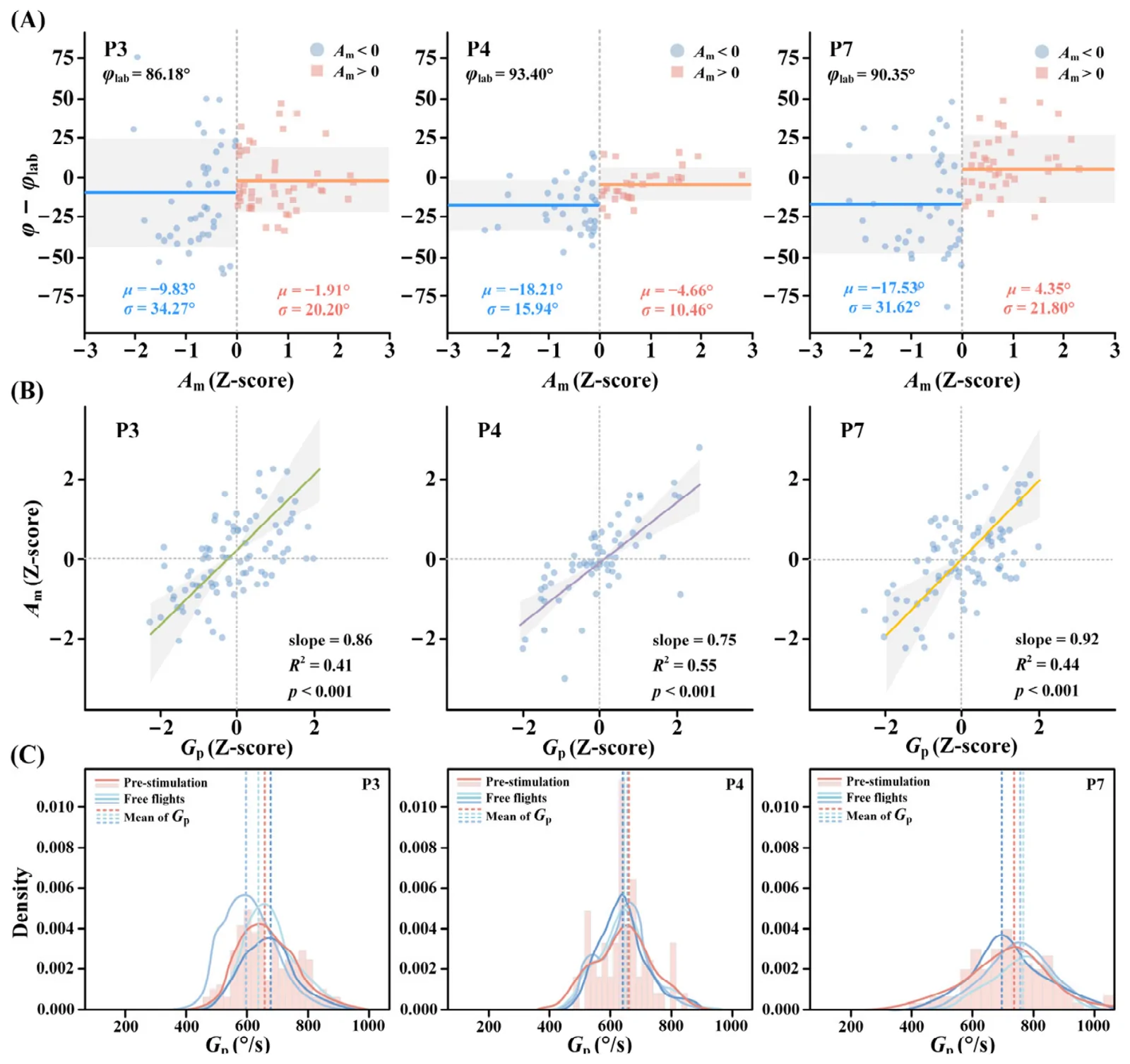
Flight states and turning-response consistency. (**A**) Turning-response deviation and dispersion across during-stimulation acceleration states; (**B**) relationship between pre-stimulation angular velocity and during-stimulation acceleration; (**C**) angular-velocity distributions during pre-stimulation and unstimulated free-flight conditions.

The preceding analyses showed that acceleration during stimulation $A_{m}$ was closely related to variations in stimulation-evoked turning responses, whereas pre-stimulation angular velocity $G_{p}$ was strongly associated with the subsequent $A_{m}$ state. Because the locomotor state during stimulation can only be observed after stimulation has begun, we next examined the relationship between the key pre-stimulation state and turning-response consistency to assess its potential value for identifying appropriate neuromodulation timing. Figure 5 shows representative results for P3, P4, and P7; results for the remaining pigeons are provided in Figures S1–S3.

Figure 5A shows the relationship between acceleration during stimulation $A_{m}$ and the deviation of free-flight turning responses from the laboratory reference level. The horizontal axis represents within-individual standardized $A_{m}$, and the vertical axis represents the difference between the free-flight stimulation-evoked turning angle and the corresponding laboratory reference turning angle. Based on the sign of standardized $A_{m}$, stimulation samples were divided into groups below and above the individual mean acceleration level. The mean difference from the laboratory reference level within each group was used to characterize systematic deviation, whereas the within-group standard deviation was used to characterize response dispersion.

Across the eight pigeons, when acceleration during stimulation $A_{m}$ was above the individual mean, the mean absolute systematic deviation of turning responses from the laboratory reference level decreased by 77.56%, while response dispersion decreased by 32.92%. These results indicate that when a pigeon’s acceleration during stimulation was above its individual mean, stimulation-evoked turning responses were, overall, closer to the laboratory reference response and showed greater consistency.

On this basis, Figure 5B further shows the relationship between pre-stimulation angular velocity $G_{p}$ and during-stimulation acceleration $A_{m}$. The two variables showed an overall positive trend: as $G_{p}$ increased, $A_{m}$ also tended to increase. When $G_{p}$ was above the individual mean, $A_{m}$ during stimulation was more likely to be above its own individual mean. Combined with the lower response dispersion associated with higher $A_{m}$ in Figure 5A, these findings indicate that pre-stimulation angular velocity provides information related to both the key locomotor state during stimulation and the consistency of subsequent turning responses.

Figure 5C further compares the distribution of pre-stimulation angular velocity $G_{p}$ with that observed during unstimulated free flight. The angular-velocity distributions under the two conditions were broadly similar, and their mean values were also similar, indicating that an individual’s reference level of angular velocity during free flight can be estimated from unstimulated conditions. Unstimulated free-flight data can therefore be used to establish an individualized angular-velocity reference level, providing a basis for identifying more appropriate neuromodulation timing for eliciting consistent turning responses.

## 4. Discussion

This study investigated the relationship between FRM microstimulation-evoked turning responses and flight state during free flight. Under relatively stable laboratory locomotor conditions, stimulation amplitude showed a clear relationship with turning response; however, when individualized stimulation parameters were held constant and applied during outdoor free flight, the turning responses still showed marked variability. Further analysis showed that locomotor state during stimulation was more closely related to the turning response than the pre-stimulation free-flight state, with during-stimulation acceleration $A_{m}$ being the most closely associated state feature. In addition, $A_{m}$ was strongly associated with pre-stimulation angular velocity $G_{p}$. When $A_{m}$ was above the individual mean, turning-response dispersion decreased by 32.92%. These findings demonstrate that FRM microstimulation-evoked turning responses are locomotor-state dependent: the effects of stimulation depend not only on stimulation parameters but also on the locomotor state present while the stimulation is being delivered.

Different responses to identical stimulation under different neural or behavioral states are a key manifestation of state-dependent neural stimulation. Previous studies have shown that the neural and behavioral effects of stimulation are modulated by the physiological state, task state, and background network activity present at the time of stimulation [19], and recent cross-species work has further demonstrated that behavioral state can alter neural responses evoked by exogenous stimulation [33]. These state-dependent differences can alter the excitability and ongoing activity of the neural circuits engaged by stimulation, thereby affecting how stimulation-evoked activity is integrated with ongoing sensorimotor processes. During movement, sensory feedback and descending motor commands are continuously integrated, while avian free flight additionally requires ongoing adjustments in wing motion, body posture, and aerodynamic output to maintain and change flight state [34]. Thus, even when stimulation parameters are identical, stimulation delivered under different locomotor states can interact with different ongoing motor processes and produce different motor responses. In the present study, stimulation amplitude was clearly related to turning response under relatively stable laboratory locomotor conditions, yet marked response variability remained when fixed individualized stimulation parameters were applied during outdoor free flight. Previous studies of avian locomotor control have primarily manipulated stimulation amplitude, frequency, duration, and inter-stimulation interval to regulate flight trajectories and turning behavior [6,9,20]. Our results further show that, once stimulation parameters are fixed, the current locomotor state remains an important determinant of the turning response during free flight.

Further analysis showed that locomotor state during stimulation was more closely related to turning response than the pre-stimulation free-flight state, with during-stimulation acceleration showing the strongest relationship with turning response. Previous work has demonstrated that FRM neural activity is closely related to flight state in pigeons: local field potentials and functional connectivity differ across accelerated takeoff, steady flight, and decelerated landing [14]. Fang et al. further reported that active turning and electrically evoked turning are associated with different patterns of high-frequency FRM oscillations, and that FRM electrical stimulation alters local neural oscillations while producing turning behavior [15]. Together, these findings indicate that FRM neural activity is related both to the current flight state and to stimulation-evoked motor responses. The stronger association observed here between during-stimulation locomotor state and turning response further suggests that analyses of FRM neuromodulatory effects should consider not only the locomotor state immediately before stimulation but also state changes occurring while stimulation is being delivered.

Notably, the key locomotor state during stimulation was not completely independent of the state present before stimulation onset. State-to-state analysis showed that, among pre-stimulation velocity, acceleration, and angular velocity, pre-stimulation angular velocity was most closely related to acceleration during stimulation. Moreover, when acceleration during stimulation was above the individual mean, turning-response dispersion decreased by 32.92%. Because during-stimulation state can only be obtained after stimulation has begun, pre-stimulation state is more suitable as information available before neuromodulation. Previous studies have shown that selecting stimulation timing according to pre-stimulation neural oscillatory power, phase, or instantaneous neural activity can alter stimulation-evoked cortical excitability, behavioral responses, and neural plasticity [35,36,37,38]. Although the present study used flight-state variables rather than neural activity to characterize pre-stimulation state, the results show a strong relationship between pre-stimulation angular velocity and the key locomotor state during stimulation. Observable pre-stimulation flight state may therefore provide useful information for subsequently identifying appropriate neuromodulation timing.

This study has several limitations. Because outdoor free-flight stimulation experiments are difficult to perform and the number of valid samples is limited, we primarily examined stimulation conditions that reliably evoked an approximately 90° turning response under laboratory conditions. Future studies should test whether the observed state dependence generalizes across different stimulation intensities and target motor responses. We currently characterized flight state using statistical features of velocity, acceleration, and angular velocity, which capture only limited temporal dynamics of wingbeats, postural adjustments, and short-term movement changes. Vertical flight movements were not analyzed separately in the present study and should be incorporated into future three-dimensional assessments of stimulation-evoked flight responses. Although experiments were conducted under low-wind conditions, minor turbulence and changes in flight altitude could still introduce transient variations in acceleration, angular velocity, and trajectory-derived velocity, thereby contributing additional variability to the extracted kinematic features. Future work combining higher-temporal-resolution kinematic measurements with simultaneous FRM electrophysiological recordings could further clarify the dynamic relationships among pre-stimulation state, neural and locomotor states during stimulation, and the final behavioral response.

## 5. Conclusions

This study analyzed the influence of locomotor state on the neuromodulatory effects of FRM microstimulation during free flight. The results demonstrate that FRM microstimulation-evoked turning responses are locomotor-state dependent: locomotor state during stimulation shows a stronger correspondence with the neuromodulatory response, whereas pre-stimulation locomotor state provides information related to the consistency of the subsequent response. Within the present sample of eight pigeons, these preliminary findings indicate that, under complex locomotor conditions, neuromodulatory effects are closely related to the locomotor state present when stimulation is delivered, providing a basis for determining neuromodulation timing and improving response stability.

## Figures and Tables

**Table 1:** Specifications of sensors integrated into the wearable miniature stimulation device.

Hardware Module	Functional Description	Sampling Rate	Mounting Position
ATGM336H	Acquisition of GPS data	10 Hz	Back-mounted
BMX160	Acquisition of attitude data	200 Hz	Head-mounted
ADG5206	Generate stimulation	/	Head-mounted

## Data Availability

The minimal dataset supporting the findings of this study has been provided with the submission. The full datasets are not publicly available because they form part of ongoing research and include data associated with studies that have not yet been published. Additional data are available from the corresponding author upon reasonable request.

## Supplementary Materials

The following supporting information can be downloaded at: https://www.mdpi.com/article/10.3390/ani16203213/s1, Figure S1. Systematic deviation and response dispersion of turning responses under different during-stimulation acceleration states. Blue and red points indicate trials with during-stimulation acceleration below and above the individual mean, respectively; horizontal lines and shaded regions indicate the corresponding mean deviation and response dispersion. Figure S2. Relationship between pre-stimulation angular velocity and during-stimulation acceleration across individual pigeons. Solid lines indicate linear fits, with the corresponding slope, R^2^, and *p* values shown in each panel. Figure S3. Probability density distributions of angular velocity under pre-stimulation and unstimulated free-flight conditions across individual pigeons. Vertical dashed lines indicate the corresponding mean angular velocities. Table S1. Stimulation amplitudes and numbers of stimulations for each pigeon. Table S2. Variance inflation factors (VIFs) of pre-stimulation and during-stimulation flight-state features. Table S3. Five-fold cross-validation performance of T-MTGP models using different covariance kernels.

## Abbreviations

The following abbreviations are used in this manuscript:

FRM	formatio reticularis medialis mesencephali
DIVA	dorsalis intermedius ventralis anterior
SGP	stratum griseum periventriculare
PoA	posterior pallial amygdala
GP	Gaussian process
MTGP	multi-task Gaussian process
NLL	negative log likelihood
SHAP	Shapley additive explanations
