# Supplementary material for: Locomotor-State Dependence of Neuromodulation During Avian Free Flight
Source: Animals (Basel). 2026 Oct 13;16(20):3213. doi: 10.3390/ani16203213 (PMC13700548; doi:10.3390/ani16203213)
Supplement: Supplementary file 1 [file animals-16-03213-s001.zip › animals-4598089-supplementary.pdf]

Supplementary Materials

Locomotor-State Dependence of Neuromodulation during Avian Free Flight

Zhuo Tang [1,2], Suchen Li [1,2], Mengmeng Li [1,2], and Zhigang Shang [1,2*]

[1]School of Electrical and Information Engineering, Zhengzhou University, Zhengzhou, China.

[2] Henan Key Laboratory of Brain Science and Brain-Computer Interface Technology, Zhengzhou, China.

[*]Correspondence: Zhigang Shang; zhigang_shang@zzu.edu.cn

This PDF file includes:

Figures S1 to S3

Tables S1 to S3

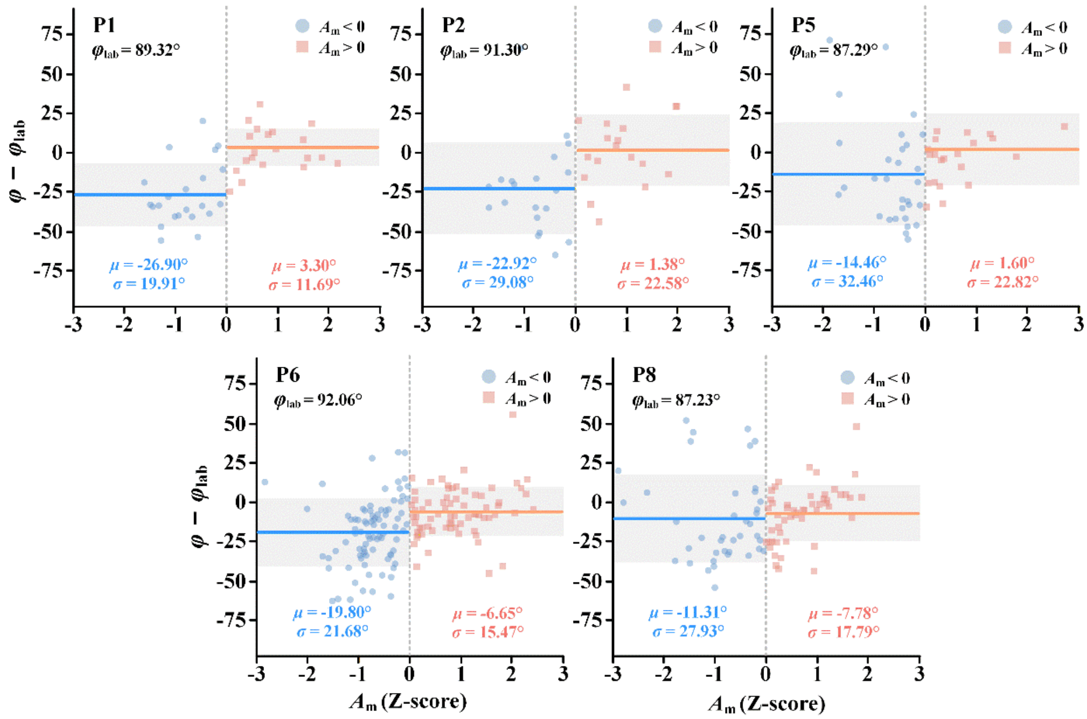

Figure S1. Systematic deviation and response dispersion of turning responses under different during-stimulation acceleration states. Blue and red points indicate trials with during-stimulation acceleration below and above the individual mean, respectively; horizontal lines and shaded regions indicate the corresponding mean deviation and response dispersion.

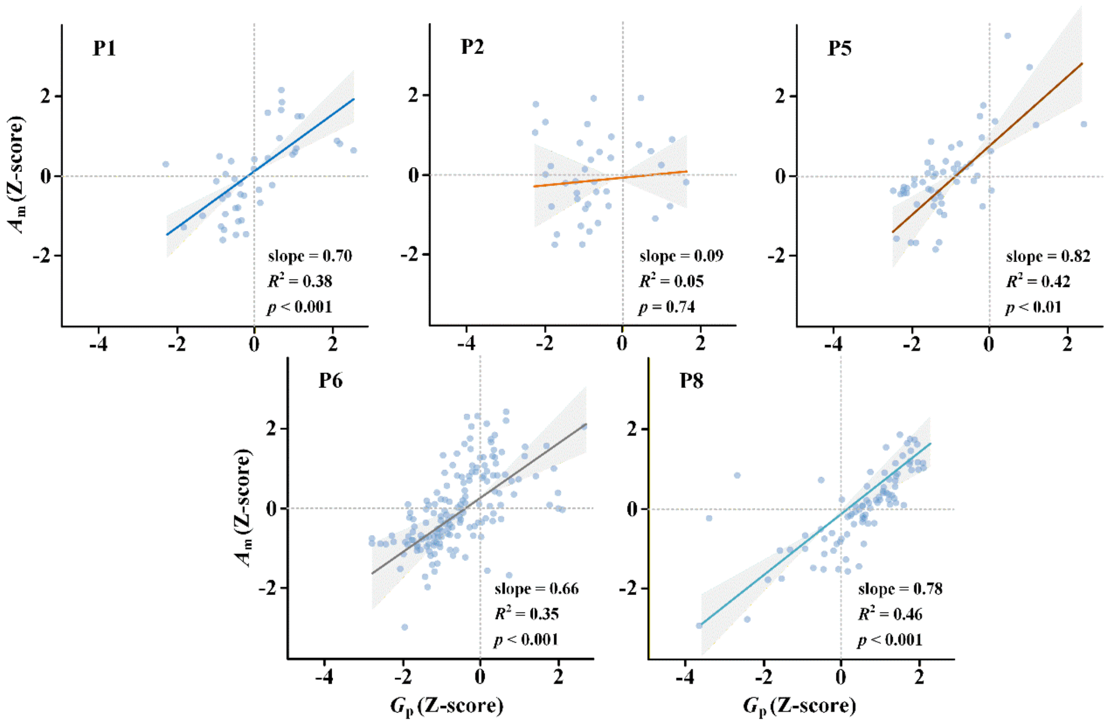

Figure S2. Relationship between pre-stimulation angular velocity and during-stimulation acceleration across individual pigeons. Solid lines indicate linear fits, with the corresponding slope, \(R^2\), and \(p\) values shown in each panel.

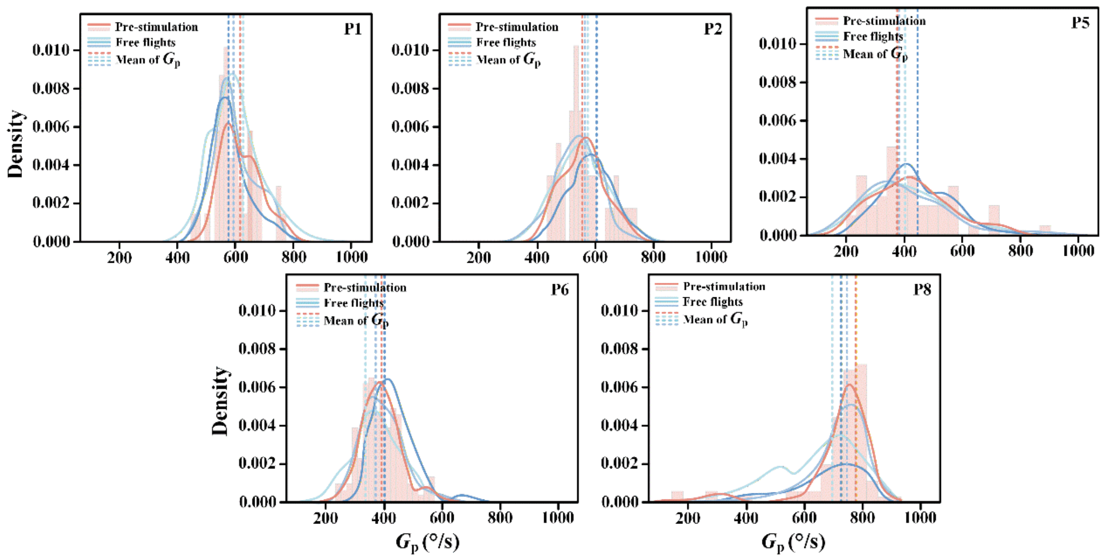

Figure S3. Probability density distributions of angular velocity under pre-stimulation and unstimulated free-flight conditions across individual pigeons. Vertical dashed lines indicate the corresponding mean angular velocities.

Table S1. Stimulation amplitudes and numbers of stimulations for each pigeon.

ID	Amp (μA)	# of stimulations	ID	Amp (μA)	# of stimulations
P1	120	42	P5	455	55
P2	540	38	P6	290	160
P3	415	90	P7	155	90
P4	160	65	P8	255	91
Total			631		

Table S2. Variance inflation factors (VIFs) of pre-stimulation and during-stimulation flight-state features.

Stage	Feature	VIF
Pre-stimulation	V_{p}	1.09
	A_{p}	1.26
	G_{p}	1.36
During-stimulation	V_{m}	1.59
	A_{m}	1.58
	G_{m}	1.01

To examine the influence of covariance-kernel selection on model performance, kernel-ablation analysis was performed using linear, exponential (Matérn-1/2), Matérn-3/2, Matérn-5/2, and RBF kernels under the same T-MTGP framework and five-fold cross-validation procedure. As shown in Table S3, the linear and exponential kernels yielded significantly higher NLL values than the RBF kernel, whereas no significant differences were observed between RBF and the Matérn-3/2 or Matérn-5/2 kernels after Holm correction for multiple comparisons. The RBF kernel achieved the lowest mean NLL for both the pre-stimulation and during-stimulation models and was therefore retained in the main analyses.

Table S3. Five-fold cross-validation performance of T-MTGP models using different covariance kernels.

Covariance kernel	Pre-stimulation NLL	During-stimulation NLL
Linear	1.021 ± 0.061**	0.586 ± 0.052**
Exponential (Matérn-1/2)	1.006 ± 0.054*	0.568 ± 0.047*
Matérn-3/2	0.967 ± 0.045	0.543 ± 0.044
Matérn-5/2	0.961 ± 0.043	0.538 ± 0.042
**RBF**	**0.960 ± 0.042**	**0.535 ± 0.043**

Values are reported as mean ± SD. Pairwise comparisons with the RBF kernel were performed using paired t-tests separately within each column, and p values were adjusted using the Holm correction for multiple comparisons. * $p < 0.05$, ** $p < 0.01$.
